# Clinical comparison of the efficiency and security of balloon dilators versus fascial dilators in percutaneous nephrolithotripsy (PCNL)

**DOI:** 10.12669/pjms.323.9281

**Published:** 2016

**Authors:** Lingbo Yang, Shuaiqi Lu, Xingtao Han, Pengtao Wei, Jinhui Yang, Tongtong Hao

**Affiliations:** 1 Prof. Lingbo Yang, MD. Department of Urology, Luoyang Central Hospital affliliated to Zhengzhou University, 471000, Luoyang, China; 2 Prof. Shuaiqi Lu, MM. Department of Urology, Luoyang Central Hospital affliliated to Zhengzhou University, 471000, Luoyang, China; 3 Prof. Xingtao Han, MD Department of Urology, Luoyang Central Hospital affliliated to Zhengzhou University, 471000, Luoyang, China; 4 Prof. Pengtao Wei, MD. Department of Urology, Luoyang Central Hospital affliliated to Zhengzhou University, 471000, Luoyang, China; 5 Dr. Jinhui Yang, MM. Department of Urology, Luoyang Central Hospital affliliated to Zhengzhou University, 471000, Luoyang, China; 6 Dr. Tongtong Hao, MM. Department of Urology, Luoyang Central Hospital affliliated to Zhengzhou University, 471000, Luoyang, China

**Keywords:** Balloon dilators, Fascial dilators, Upper urinary tract calculi, Security, Efficiency

## Abstract

**Objective:**

To compare the efficiency and security of the balloon dilators versus fascial dilators in percutaneous nephrolithotripsy (PCNL), We compared the difference of intraoperative and postoperative parameters of patients using these two different methods of expansion and having no significant statistic differences in peroperative parameters.

**Methods:**

This is a retrospective analysis of 134 patients undergoing PCNL with upper urinary calculi from January 2012 to January 2014 in Luoyang Central Hospital affiliated to Zhengzhou University. These patients meeting the inclusion criteria were divided into two groups: the group of balloon dilators (group A) and the group of fascial dilators (group B). Two groups were compared for success rate of first expansion, clearance of stone, duration of surgery, intraoperative hemorrhage, blood transfusion rate, postoperative hospitalization and the incidence of complications.

**Result:**

In Group A, a total of 91 patients (51 men and 40 women, mean age 51.22±8.96 years, ranged from 28 to 68 years, the calculi maximum diameter from 0.9 to 4.5cm, 28 cases with a history of gravel, mean Body mass index 24.20±2.34, 73 cases with hydronephrosis and 26 cases with underlying diseases such as hypertension, diabetes and the like) undergoing PCNL were retrospectively reviewed. Similarly, In Group B, a total of 43 patients (28 men and 15 women, mean age 49.64±10.62 years, ranged from 15 to 70 years, the calculi maximum diameter from 1.1 to 5.2cm, 18 cases with a history of gravel, mean Body mass index 24.40±2.70, 38 cases with hydronephrosis and 14 cases with underlying diseases such as hypertension, diabetes and the like) undergoing PCNL were retrospectively reviewed. Our results showed that there was a statistically significant better outcome in Group A than in Group B in terms of success rate of first exploration, duration of operation, intraoperative hemorrhage, postoperative hospitalization and the incidence of complications. Additionally, there was no statistically significant difference with respect to clearance of stone and incidence of blood transfusion in the two groups.

**Conclusion:**

Balloon dilators had shorter operation time, less bleeding, higher success rate of first expansion, less postoperative complications and shorter postoperative hospitalization than fascial dilators in PCNL.

## INTRODUCTION

Upper urinary tract calculi disease is one of the most common urological disorders recognized since ancient times, with a prevalence of approximately 2–3% in the general population. What is worse, the rate of incidence of renal calculi associated with renal inadequacy is about 12.7%.^1^ Therefore, it is particularly important to diagnose and cure early.

Renal stones were classically removed by open surgery, but the advent of minimally invasive, endoscopic techniques, extra-corporeal shock wave lithotripsy (ESWL) and retrograde intrarenal surgery (RIRS) have almost replaced the classically performed open surgery for the removal of upper urinary tract calculi. However, the technique of percutaneous nephrolithotripsy (PCNL) is now considered as the standard treatment for large and complex renal stones.^2^ The key of PCNL is to establish the operation channel containing the two methods: balloon dilatation and fascial dilatation. To date, there is no clear evidence as to which method can bring better outcomes in PCNL. Our objective was to compare the efficiency and security of the balloon dilators versus fascial dilators in percutaneous nephrolithotripsy (PCNL), We retrospectively analyzed the clinical data of 134 patients with using these two different methods of expansion and having no significant statistic differences in peroperative parameters and then compared the difference of intraoperative and postoperative parameters of patients.

## METHODS

This study selected 134 cases of patients with upper urinary calculi from Luoyang Central Hospital affiliated to Zhengzhou University from January 2012 to January 2014. These patients meeting the inclusion criteria were divided into two groups: the group of balloon dilators (Group A) and the group of fascial dilators (Group B), which used Cybersonics double-catheter system to fragment the stones. All the data relating to baseline characteristics of the participants are mentioned under the supporting table A and B. These patients with urinary tract infection were first given anti-inflammatory treatment. The operation was considered when blood and urine routine test returned to normal. There was no statistically significant difference (P>0.05) in clinical characteristics of patients in the two groups. Therefore, the effects of two methods were comparable (Table-I).

**Table-I:** Demographic and clinical characteristics of patients (x±s).

*Factor*	*Group A (N=91)*	*Group B (N=43)*	*P*
Age(year)	51.22±8.96	49.64±10.62	0.233
Male/Female	51/40	28/15	0.319
Maximum stone diameter (cm)	2.39±0.82	2.37±0.97	0.969
Mean BMI	24.20±2.34	24.40±2.70	0.663
Hydronephrosis, yes/no	73/18	38/5	0.139
Underlying diseases, yes/no	26/65	14/29	0.634
History of grave, n	28	18	0.207
Urinary tract infection, n	69	29	0.307

There was no statistically significant difference (P>0.05) in clinical characteristics of patients in the two groups. Therefore, the effects of two methods are comparable.

### The inclusion criteria

(1) All the patients were diagnosed definitely as unilateral upper urinary tract calculi before operation with a plain film X-rays and/or intravenous pyelography and/or computer tomography (CT) scan. (2) Patients had normal cardiovascular and pulmonary function or patients had some underlying diseases such as hypertension, diabetes, arrhythmia and the like which must be controlled actively before operation. (3) Patients with severe underlying diseases including blood coagulation dysfunction, renal dysfunction, severe urinary tract infection and obesity (BMI≥30) would be rejected into the study. (4) All surgeries were performed by the same surgeon during the same time period.

### Equipment and Instruments

It included an 18-gauge coaxial needle, storz cystoscopy and F20 nephroscope, fascial dilators (Cook Medical Inc. USA), X-ForceN30 nephrostomy, balloon dilators Catheter(BCR Inc. USA), F9.8 olympus ureteroscope, cybersonics double catheter system, Ultrasound (Hitachi) instrument with transducer frequency 3.5 MHz, Fluoroscopic table, 0.032-inch floppy- tipped guide wire and so on.

### Preoperative preparation

Imaging examinations should be taken to ensure the diagnosis before operation. Positive preoperative preparation including blood and urine routine, the functions of the liver and kidney, coagulation and other routine test must be implemented. These basic diseases just like hypertensive and diabetes must be actively controlled preoperatively. Smoking and drinking were strictly prohibited for a week prior to the operation. Preoperative fasting was advised for 12 hours. One day ahead of the schedule for preoperative Intestinal preparation.

### Statistical Methods

All data were expressed as the mean±SD. Qualitative data were analyzed by using the X^2^, measurement data were compared by means of T test (SPSS 17.0, SPSS, Chicago). P<0.05 was considered to be statistically significant.

**Fig.1:**
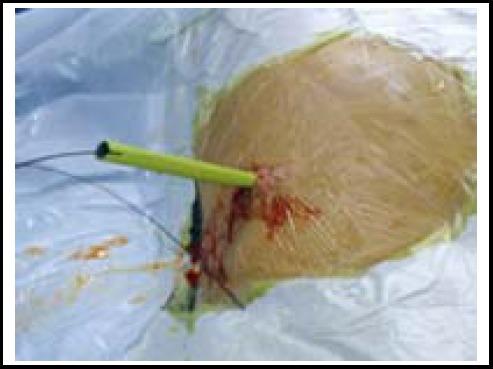
The group of balloon dilator.

**Fig.2:**
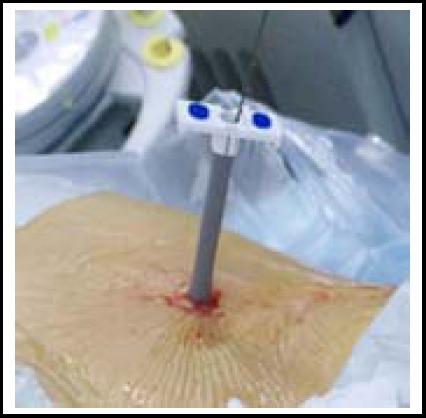
The group of fascial dilator.

The entire procedure was performed on the Fluoroscopic Table with the patient under general anesthesia. A F5 open-ended ureter catheter was firstly performed for preparing for the formation of a man-made hydronephrosis^3^ and then the patient was repositioned to the prone position. An 18-gauge coaxial needle was introduced into the targeted calyx along the 12th rib or 11th intercostal space, which provided the best access to the targeted calyx and minimized the surgical risk.^4^ A 0.032-inch floppy-tipped guidewire was passed through into the collecting system. A working channel was established using the X-Force N30 nephrostomy, balloon dilators (Fig.1) or fascia dilators from F8 to F24 along the guidewire (Fig.2). Subsequently, F20 nephroscope and the cybersonics double-catheter system were used to fragment the renal stone. Finally, a clamped F20 Foley catheter was placed as a nephrostomy tube, which was opened within 24h and was removed when the color of the extravasation changed to clear gradually. Besides, we routinely setted a double J tube into the ureter and removed it about one month postoperatively.^5^ Counterchecked blood routine and KUB to ensure whether anemia and residual stones occurred after one week postoperatively. Patients were considered stone free when no stone >5 mm was visualized.

The compared parameters were as follows: the success rate of first expansion, stone-free rate, duration of operation(minutes), intraoperative hemorrhage, postoperative hospitalization(d), blood transfusion rate and incidence of complications (on-going hematuresis, postoperative fever, leakage of urine, thoracic or abdominal organ injury, septic shock, renal embolization, nephrectomy, death and so on).

**Fig. 3 and 4:**
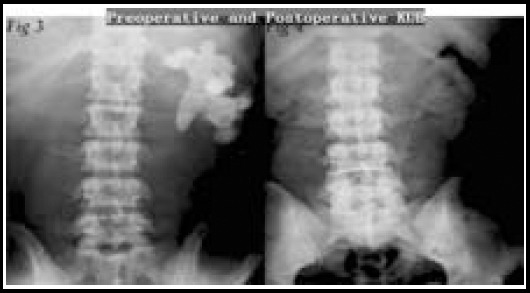
Preoperative and postoperative KUB.

In our series, we estimated roughly the intraoperative hemorrhage. The amount of bleeding=(preoperative hemoglobin-postoperative hemoglobin) /preoperative hemoglobin x total blood volume.^6^ Besides, In case of postoperative hemoglobin<70g/L, we would transfuse same-type red cell suspension into the body of the patient. If the postoperative hemoglobin was in between 70g/L and 100g/L. Simultaneously, the patient showed palpitations, thirst and other performances of shock, we would consider to transfuse some red blood cell suspension or frozen plasma. As shown below, the KUB of peroperation and postoperation showed stone clearance situation (Fig. 3 and 4).

## RESULTS

The comparison of clearance of stone, blood transfusion rate, success rate of first expansion and incidence of complications in group A and group B, were observed. The difference of clearance of stone between in group A and in group B was considered to be not statistically significant (78/91 vs 32/43, P=0.111>0.05). Similarly, the difference of blood transfusion rate was of no statistical significance (2/91 vs 2/43, P=0.593>0.05). The success rate of first expansion was significantly higher (89/91 vs 38/43, P=0.035<0.05) in group A than in group B. Compared to group B, the incidence of complications was significantly lower in group A(11/91 vs 13/43, P=0.011< 0.05).(Table-II)

**Table-II:** The comparison of efficiency and security in the two groups.

*Parameters*	*Group A(N=91)*	*Group B(N=43)*	*P*
Clearance rate of stone,%	78/91	32/43	0.111
Blood transfusion rate,%	2/91	2/43	0.593
Success rate of first expansion,%	89/91	38/43	0.035
Incidence of complications,%	11/91	13/43	0.011
Duration of operation(min)	71.95±39.95	93.14±51.40	0.002
Intraoperative hemorrhage(ml)	91.76±78.44	136.51±106.55	0.002
Postoperative hospitalization(d)	7.63±1.63	8.26±1.35	0.019

The results of operation in group A were superior to the one in group B, but the difference of efficiency in group A and group B was not statistically significant.

### The comparison of duration of operation, intraoperative hemorrhage and postoperative hospitalization in group A and group B

The duration of operation was significantly shorter (71.95±39.95minutes vs 93.14±51.40 minutes, P=0.002<0.05) in group A than in group B. Compared to group B, intraoperative hemorrhage was much lower in group A(91.76±78.44 vs 136.51±106.55, P=0.002<0.05). Just like the two parameters mentioned above, the postoperative hospitalization in group A was superior to the one in group B(7.63±1.63 vs 8.26±1.35, P=0.019<0.05)(Table-II).

### The complications in group A and group B

The complications was significantly higher in group B than group A. Postoperative fever occurring in nine patients of group A and 10 patients of group B was the most common complication. What’s worse, one patient in group A and one patient in group B developed septic shock (Table-III). The complication rate was higher in the fascial dilation group than the balloon dilation. But this is driven by a higher rate of fever, even nephrectomy.

**Table-III:** Complications in two groups.

*Complications*	*Group A (N=91)*	*Group B (N=43)*
Hematuresis	0	1(1/43)
Fever	9(9/91)	10(10/43)
Septic shock	1(1/91)	1(1/43)
Nephrectomy	0	1(1/43)
Embolization	1(1/91)	0
Pneumothorax	0	0
Abdominal organ injury	0	0
Death	0	0
Others	0	1

Although the complication rate is higher in the fascial dilation group, this is driven by a higher rate of fever, even nephrectomy. But there was no obvious difference on peroperation infection in the two groups.

## DISCUSSION

The incidence of upper urinary tract calculi present a burgeoning trend in recent years. Therefore, strengthening the prevention and treatment of stone become more crucial. PCNL is a well-established treatment option for patients with large and/or complex renal calculi among the numerous treatment ways of calculus removal.^7^ A correct access tract through the collecting system and its proper dilators are key procedures in PCNL.^8^ Because the PCNL channel is directly related to intraoperative and postoperative parameters. The ideal PCNL channel should have the shortest distance away from the calculi and enter directly into the fornix of the targeted calyx and inspect other calyxes conveniently.^9^ Balloon dilators and fascial dilators were widely used for channel dilatations in PCNL.^10^ The working tract is established by ultrasound-guided or X-ray. In our study, we chose ultrasound-guided to localize and dilate the PCNL channel. Ultrasound have more advantages than X-ray such as cheaper, absence of ionizing radiation and real-time visualization of surrounding structures. What’s more, no need for contrast agent is another advantage of ultrasound guidance.^11^ In our study, we chose ultrasound-guided to localize and dilate the PCNL channel.

As shown in Table-II, the success rate of first expansion (89/91 vs 38/43, p=0.035<0.05) was significantly higher in group A than in group B. Two patients from group A and 5 patients from group B suffered a failure in the first expansion. In group A, one patient with a history of ipsilateral PCNL three months ago failed in the first expansion because of surgical scar. The other was the patient with complete staghorn kidney stones, which possessed a very small renal pelvis space and impacted guidewire implantation and balloon dilatation catheter closing to the effective position. Both the patients were used a F10 fascial dilator to dilate the working tract firstly and then used balloon dilatation catheter to dilate the channel. Fortunately, the channel was established successfully at last, which confirmed the effect of the combination of fascial dilators and balloon dilators.^12^ Similarly, the failure of five patients in group B were due to the sliding out of guidewire, which led to the loss of the passage. We added another channel to complete the PCNL channel successfully. In conclusion, 100% of the patients in group A and 88.32% of patients in group B could be rendered stone free with a single tract. On this point, we can understood that balloon dilators catheter had more obvious advantage than fascial dilators in PCNL.

The amount of intraoperation hemorrhage (91.76±78.44ml vs 136.51±106.55ml, P=0.002<0.05) and duration of operation (71.95±39.95minutes vs 93.14±51.40min, p=0.002<0.05) were statistically significantly lower in group A than in group B. Even the incidence of complications (11/91 vs 13/43, p=0.011<0.05) and hospital stay (7.63±1.63d vs 8.26±1.35d, p=0.019<0.05) were lower in group A than in group B. This result was consistent with Turna’ report.^13^ All the aforementioned data are presented in Table-II. The disadvantages of fascial dilators were bleeding in the dilating process, the loss of passage and even the perforation of the collection system. The main disadvantage was that it was difficult to control the depth and pressure exerted during dilation. Balloon dilators just needed one-step to complete the working channel. it was a transverse and homogeneous dilation, which imposed an unremitting pressure on small vessels around and reduced the possibility of bleeding and even rupture. Additionally, the perfect combination of sheath and pressurized balloon decreased the probability of renal hemorrhage. All the advantages reflected in balloon dilators were responsible for less mean bleeding, shorter duration of operation, smaller trauma, shorter time of recovery and lower risk of postoperative complications. This result was consistent with that reported by Aminsharifi.^14^

There was no statistically significant difference between the two groups in terms of clearance of stone (78/91 vs 32/43, p=0.111>0.05) and the incidence of blood transfusion (2/91 vs 2/43, p=0.593>0.05). Given the less bleeding and less influence on the endoscopic view in group A, we should have higher clearance of stone rate and lower incidence of blood transfusion in theory. But we did not find the statistically significant difference with respect to clearance of stone and incidence of blood transfusion in the two groups, the reason of which might be related to smaller clinical samples and stone-related parameters (opacity, number, burden and so on).

The most severe complication in our series was bleeding, which happened in one patient of group A and two patients of group B. The patient, in group A, had a history of ipsilateral PCNL three months ago. Surgical scar decreased renal activity distinctly so that we damaged the renal parenchyma and encounted renal nephrostomy passage continuing bleeding in the process of PCNL. We terminated the operation firstly, placed a F20 Foley Catheter into the same nephrostomy passage and then gave a hemostatic by intramuscular injection. Unfortunately, the bleeding continued. After discussion with the dependents of the patient, we gave the patient embolization of renal artery branch, and then the bleeding stopped. One month later, we fragmented the residual calculi successfully with RIRS.

The abdominal cavity and retroperitoneum of one patient were penetrated isotonic solution through the working tract in group B, which caused abdominal distension, espiratory resistance increasing. At last, we terminated the operation and indwelt closed drainage of abdominal cavity. One month later, we fragmented the residual calculi successfully with ESWL. The other was a complete staghorn calculi with two tracts, which took us long time to fragment the stones. We damaged the renal parenchyma causing the kidney bleeding. We imbedded a F20 Foley Catheter into the working channel to compress bleeding spots. Fortunately, the bleeding stopped. But when we removed the F20 Foley Catheter at approximately 5 days postoperation, the bleeding started again. Unfortunately, the hemorrhagic situation changed almost nothing. After discussion with the dependents of the patient, the nephrectomy was performed.

The most common complication in our series was postoperative fever, which occurred in 9 patients of group A and 10 patients of group B. what’s worse, one patient in group A and one patient in group B developed septic shock and were given anti-shock therapy. The longer the duration of operation, the more the probability of postoperative fever. Referring to the latest relevant literatures and combining with our own experience, we believe that the reason of fever after operation were the following points: Firstly, the hidden bacterium in infectious stones retrograded into the blood. Secondly, the postoperative renal fistula, double-J stent and catheter are foreign bodies, which cause fever easily and even more obvious in inadequate drainage. Thirdly, high pressure in pelvises during operation. Fourthly, lack of pre-operation anti-inflammatory therapy besides neglecting the duration of surgery and the amount of irrigation fluid. As a postoperative outcome, the incidence of postoperative fever (9/91 vs 10/43, p=0.038<0.05) was statistically significantly lower in group A than group B. The reason might be related to the point that the group A had shorter duration of operation and less bleeding than the group B. The result was consistent with the Akin Y.^15^ As a result of our study, there was no complications such as pneumothorax, intestinal injury and so on, the reason of which was the application of balloon dilators in PCNL.

### Limitations of the study

The retrospective design, during which the same surgeon decided how to dilate the working channel. Yet another drawback was ignoring the stone burden effect on the two groups. What’s more, the number of cases in the study is comparatively smaller.

## CONCLUSION

Balloon dilators have shorter operation time, less bleeding, higher success rate of first expansion, less postoperative complications and shorter postoperative hospitalization than fascial dilators in PCNL. But the study is under-powered, design is retrospective, the number of cases in the study is comparatively smaller, which result in lack of enough confidence and these conclusions cannot be generalized.

## References

[1] Akman T, Binbay M, Aslan R, Yuruk E, Ozgor F, Tekinarslan E (2012). Long-term outcomes of percutaneous nephrolithotomy in 177 patients with chronic kidney disease:a single center experience. J Urol.

[2] Farhan M, Nazim SM, Salam B, Ather MH (2015). Prospective evaluation of outcome of percutaneous nephrolithotomy using the ‘STONE’ nephrolithometry score:A single-centre experience. Arab J Urol.

[3] Yuanzhong D, Xin G, Bo T, Gui-rong C, Jie-lin T (2006). Treatment of renal calculi with minimally invasive percutaneous nephrolithotomy guided by ultrasound. J Chongqing Med Uni.

[4] McDougal W Scott, Alan J Wein, Louis R Kavoussi, Andrew C Novick (2012). Campbell-Walsh Urology.

[5] Chang QS, Li H, Zhu YS, Ma N, Kong DZ (2014). A comparison of application value of different channel setup ways in percutaneous nephroscope lithotripsy. Anhui Med Pharmaceutical J.

[6] Yan X, Guo HQ, Li XG, Gan WD, Zhang SW, Yang Y (2008). Factors affecting blood loss during mini-percutaneous nephrolithotomy using ureteroscope and pneumatic intracorporeal lithotripsy. Chin J Urol.

[7] Ferakis N, Stavropoulos M (2015). Mini percutaneous nephrolithotomy in the treatment of renal and upper ureteral stones:Lessons learned from a review of the literature. Urol Ann.

[8] Park S, Pearle MS (2006). Imaging for percutaneous renal access and Management of renal calculi. Urol Clin North Am.

[9] Mei H, Chen LW, Gao L (2007). Urologyical Surgery. People’s medical publishing house.

[10] Ozok HU, Sagnak L, Senturk AB, Karakoyunlu N, Topaloglu H, Ersoy H (2012). A comparison of metal telescopic dilators and Amplatz dilators for nephrostomy tract dilation in percutaneous nephrolithotomy. J Endourol.

[11] Fu YM, Chen QY, Zhao ZS, Ren MH, Ma L, Duan YS (2011). Ultrasound-guided minimally invasive percutaneous nephrolithotomy in flank position for management of complex renal calculi. Urology.

[12] De Lisa A, Caddeo G (2010). PCNL:tips and tricks in targeting, puncture and dilation. Arch Ital Urol Androl.

[13] Turna B, Nazli O, Demiryoguran S, Mammadov R, Cal C (2007). Percutaneous nephrolithotomy:variables that influence hemorrhage. Urology.

[14] Aminsharifi A, Alavi M, Sadeghi G, Shakeri S, Afsar F (2011). Renal parenchymal damage after percutaneous nephrolithotomy with one-stage tract dilation technique:a randomized clinical trial. J Endourol.

[15] Akin Y, Basara I, Yucel S, Gulmez H, Ates M, Bozkurt A (2013). Is tubeless PCNL really less injurious than standard in the midterm?. J Endourol.

